# Neuroprotective effects of novel antiepileptic drug lacosamide via decreasing glial activation in the hippocampus of a gerbil model of ischemic stroke

**DOI:** 10.1186/2197-425X-3-S1-A776

**Published:** 2015-10-01

**Authors:** JH Cho, CW Park, TG Ohk, MC Shin, YS Kim, MH Won

**Affiliations:** Kangwon National University, Emergency Medicine, Chuncheonsi, Korea, Republic of; Kangwon National University, Neurobiology, Chuncheonsi, Korea, Republic of

## Introduction

Lacosamide, a novel antiepileptic drug, has been discovered to have some beneficial effects beyond its effectiveness.

## Objectives and methods

In the present study, we examined the neuroprotective effect of lacosamide against ischemic damage in the hippocampal CA1 region following 5 min of transient cerebral ischemia in gerbils using H & E staining, NeuN immunohistochemistry and F-J B staining.

## Results

The results showed that pre- and post-treatment with 25 mg/kg lacosamide significantly protected neuronal death from transient cerebral ischemic injury. Many H&E positive cells, NeuN-immunoreactive neurons and a few number of F-J B-positive cells were found in the stratum pyramidale of the CA1 region in the lacosamide-treated-ischemia-operated groups compared with those in the vehicle-treated-ischemia-operated group. In addition, the treatment with 25 mg/kg lacosamide markedly attenuated the activation of astrocytes and microglia in the ischemic CA1 region.

## Conclusions

In brief, these results indicate that both pre- and post-treatment with lacosamide can protect CA1 pyramidal neurons from transient cerebral ischemic injury in the hippocampus and the neuroprotective effect of lacosamide may be related with decreasing the activation of glial cells in the ischemic CA1 region.

